# AF and in-hospital mortality in COVID-19 patients

**DOI:** 10.1016/j.hroo.2023.10.004

**Published:** 2023-10-16

**Authors:** Irum D. Kotadia, Maria Dias, Caroline Roney, Richard A. Parker, Robert O’Dowling, Neil Bodagh, José-Alonso Lemus-Solis, Daniel O’Hare, Iain Sim, David Newby, Steven Niederer, Jonathan Birns, Peter Sommerville, Ajay Bhalla, Mark O’Neill, Steven E. Williams

**Affiliations:** ∗ Biomedical Engineering and Imaging Sciences, King’s College London, London, United Kingdom; † Centre for Cardiovascular Science, University of Edinburgh, Edinburgh, United Kingdom; ‡ Stroke Medicine, Guy’s and St Thomas’ NHS Foundation Trust, London, United Kingdom

**Keywords:** COVID-19, Atrial fibrillation, COVID-19 and cardiovascular complications, COVID-19 and arrhythmia, SARS-CoV-2

## Abstract

**Background:**

There are conflicting data on whether new-onset atrial fibrillation (AF) is independently associated with poor outcomes in COVID-19 patients. This study represents the largest dataset curated by manual chart review comparing clinical outcomes between patients with sinus rhythm, pre-existing AF, and new-onset AF.

**Objective:**

The primary aim of this study was to assess patient outcomes in COVID-19 patients with sinus rhythm, pre-existing AF, and new-onset AF. The secondary aim was to evaluate predictors of new-onset AF in patients with COVID-19 infection.

**Methods:**

This was a single-center retrospective study of patients with a confirmed diagnosis of COVID-19 admitted between March and September 2020. Patient demographic data, medical history, and clinical outcome data were manually collected. Adjusted comparisons were performed following propensity score matching between those with pre-existing or new-onset AF and those without AF.

**Results:**

The study population comprised of 1241 patients. A total of 94 (7.6%) patients had pre-existing AF and 42 (3.4%) patients developed new-onset AF. New-onset AF was associated with increased in-hospital mortality before (odds ratio [OR] 3.58, 95% confidence interval [CI] 1.78-7.06, *P <* .005) and after (OR 2.80, 95% CI 1.01-7.77, *P <* .005) propensity score matching compared with the no-AF group. However, pre-existing AF was not independently associated with in-hospital mortality compared with patients with no AF (postmatching OR: 1.13, 95% CI 0.57–2.21, *P =* .732).

**Conclusion:**

New-onset AF, but not pre-existing AF, was independently associated with elevated mortality in patients hospitalised with COVID-19. This observation highlights the need for careful monitoring of COVID-19 patients with new-onset AF. Further research is needed to explain the mechanistic relationship between new-onset AF and clinical outcomes in COVID-19 patients.

**Key Findings**

▪ New-onset atrial fibrillation (AF) is the most common cardiac arrhythmia complication in patients hospitalized with COVID-19.
▪ Pre-existing AF is not associated with all-cause in-hospital mortality in patients with COVID-19 after adjusting for age, sex, race and preadmission CHA_2_DS_2_-VASc (congestive heart failure, hypertension, age ≥75 years, diabetes mellitus, prior stroke or transient ischemic attack or thromboembolism, vascular disease, age 65–74 years, sex category) score.
▪ Patients with new-onset AF in the context of COVID-19 have an increased risk of all-cause in-hospital mortality, need for mechanical ventilation, and critical care admission.
▪ Patients with new-onset AF in the context of COVID-19 should be closely monitored for acute deterioration and need for escalation of care.

## Introduction

Over 750 million cases of COVID-19 have been reported worldwide.^1^ The World Health Organization has reclassified COVID-19 from pandemic to endemic status, indicating that it is likely to remain an ongoing global issue.^2^ With endemicity remains the ability for viral evolution that can be rapid and give rise to more virulent strains as occurred with the Delta and Omicron variants, highlighting the need for ongoing research of the COVID-19 process.

Atrial fibrillation (AF) has been observed as the most common arrhythmia in the context of COVID-19, with the prevalence rate reportedly as high as 16.5% and linked to hemodynamic compromise in patients with severe illness.^3^ Over 35 studies have assessed clinical outcomes in patients with AF and COVID-19; however, the majority have either grouped all AF patients together or assessed new-onset AF alone.^4^, ^5^, ^6^, ^7^, ^8^, ^9^, ^10^, ^11^ Only 4 studies have described the clinical characteristics and outcomes of pre-existing AF and new-onset AF in patients with COVID-19.^7^^,^^12^, ^13^, ^14^ Of these, 3 studies have employed manual chart review in samples of 160 to 673 patients but present conflicting data as to whether new-onset AF is an independent marker of mortality in patients with COVID-19.^7^^,^^12^^,^^13^ All of these studies include small sample sizes, and larger studies are therefore needed to validate these findings.^15^^,^^16^

Critically, only one large study has been performed.^14^ This study was reliant on natural language processing methods for AF categorization. While machine learning techniques have shown promise in rapidly assessing large quantities of data, their accuracy has been questioned.^15^ Furthermore, a previous study comparing incidence, predictors, and outcomes of patients with AF in COVID-19 highlighted significantly higher rates of AF diagnosis using expert physician manual chart review compared with automated data collection.^10^ These findings demonstrate the clear need for manual data collection above automatic methods in this subject area.

The primary aim of this study was therefore to compare patient outcomes in those with pre-existing AF, new-onset AF, and sinus rhythm when hospitalized with COVID-19 infection, using manual chart review. The secondary aim was to evaluate predictors of new-onset AF in patients with acute COVID-19 infection.

## Methods

### Study design and population

**Figure 1:**
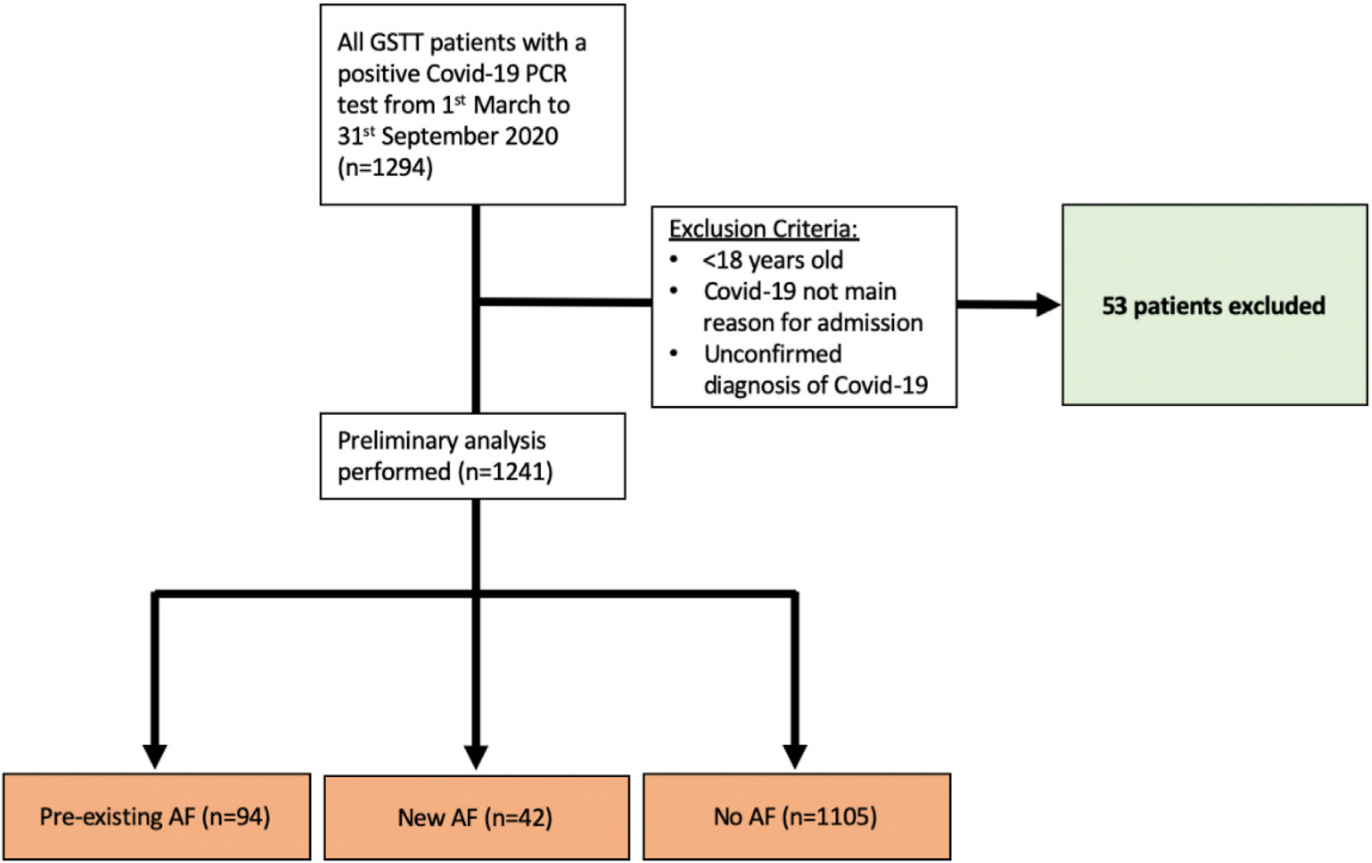
Study profile. AF = atrial fibrillation; GSTT = Guy’s and St Thomas’ Hospital; PCR = polymerase chain reaction.

A single-center, retrospective cohort study was performed including all adult patients with a completed attendance/admission to Guy’s and St Thomas’ Hospital who tested positive for SARS-CoV-2 by reverse transcription polymerase chain reaction on at least 1 occasion over the period of March 1 to September 31, 2020 (Figure 1). Ethical approval was granted by Health Research Authorities and the South London Research Ethics Committee (REC: 20/SC/0292). Patients were excluded if they were <18 years of age on admission or had an unconfirmed COVID-19 diagnosis (eg symptoms consistent with COVID-19 in the absence of a positive test result), or where COVID-19 was not the primary reason for admission.

The following data were manually extracted from electronic healthcare records by expert physician chart review: patient demographics (age range [18–27, 28–37, 38–47, 48–57, 58–67, 68–77, 78–87, 88–97, and >97 years], sex, ethnicity), clinical data (medical and social history, clinical status on admission, clinical progress, investigations, treatment delivery), and clinical outcomes (length of hospital/intensive care unit stay, maximum level of care required, oxygen requirement, need for noninvasive/invasive ventilation, discharge destination, hospital mortality). Patients were grouped according to AF status: new-onset AF, pre-existing AF, and no AF. New-onset AF was defined as any diagnosis of atrial fibrillation in a patient not previously diagnosed with atrial fibrillation. Pre-existing AF included all patients with a previous diagnosis of AF, of any subtype, including those who may have been in sinus rhythm at the time of admission. Patients in the no-AF group had neither new or pre-existing AF.

The primary outcome was all-cause inpatient mortality. Secondary outcomes were intensive care admission, requirement for mechanical ventilation, stroke, and systemic thromboembolism.

### Definitions

A positive COVID-19 diagnosis was made on nasopharyngeal or oropharyngeal swabs sent for COVID-19 ribonucleic acid testing.

### Patient and public involvement

This study was performed under the Control of Patient Information Notice declared by the Secretary of State for Health and Social Care to support the national response to COVID-19. Informed patient consent was therefore not obtained. The study conforms to the principles outlined in the Declaration of Helsinki. Verbal and written feedback was received from members of the public and incorporated into the study design.

### Statistical analysis

Statistical analyses were performed using RStudio (version 1.3.1093). Descriptive statistics were used to characterize the study population. Normally distributed continuous variables were expressed as mean ± SD, and 2-sample *t* tests were performed to compare groups. Tests for normality and homogeneity in variances were performed using the Shapiro-Wilk test and *F* test, respectively. Nonparametric data were expressed as median (interquartile range), and the Mann-Whitney *U* test was performed for group comparison.

Unadjusted comparisons were made using chi-square test (or Fisher’s exact test where sample size was <5) for categorical data and included all patients from the original dataset. Adjusted comparisons were performed following propensity score matching between pre-existing/new AF and no-AF groups. Propensity score matching was performed using the MatchIt package^16^ in RStudio to implement 1:1 nearest-neighbor matching using propensity scores generated from a logistic regression model. Remaining patients in the no-AF group that were not matched were excluded from further analysis. The following covariates were included in the matched design: age group, sex, race, and preadmission CHA_2_DS_2_-VASc (congestive heart failure, hypertension, age ≥75 years, diabetes mellitus, prior stroke or transient ischemic attack or thromboembolism, vascular disease, age 65–74 years, sex category) score. Preadmission CHA_2_DS_2_-VASc score has been used as a metric for comorbidity status. Covariate balance was assessed before and after matching. Matched analysis was performed using conditional logistic regression. All covariates were adjusted for in the matched analysis. Inpatient survival probability was measured using Kaplan-Meier analysis. A log rank test was conducted to determine if there were differences in the survival distributions for the different types of intervention. Pairwise comparison was performed to test statistical significance between groups. Post hoc analysis was performed with Bonferroni correction. All tests were 2-sided, and *P <* .05 was considered statistically significant.

## Results

From March 1, 2020, to September 31, 2020, there were 1294 patients diagnosed with COVID-19 at our institution. Of these, 1241 patients were admitted with a primary diagnosis of COVID-19 and included in this study (Table 1). The median age range was 58 to 67 years of age, and 730 (59%) patients were male. Intensive care unit admission was required in 339 (26%) patients, while 272 (22%) patients required mechanical ventilation.

**Table 1:** Demographics and clinical baseline characteristics of patients with COVID-19 infection, stratified by diagnosis of atrial fibrillation

	Pre-existing atrial fibrillation (n = 93)	New atrial fibrillation (n = 42)	No atrial fibrillation (n = 1106)	*P* value
Age, y	78-87	68-77	58-67	<.005
Male	49 (53)	22 (52)	659 (60)	.25
Race				.24
Asian	5 (5)	1 (2)	32 (3)	
Black	6 (6)	3 (7)	87 (8)	
White	43 (46)	22 (52)	434 (39)	
Other minority ethnic	30 (32)	7 (17)	352 (32)	
Unknown	10 (11)	9 (21)	200 (18)	
Median body mass index, kg/m^2^	25-29.9	25-29.9	25-29.9	.99
Comorbidities				
Hypertension	22 (24)	9 (21)	147 (13)	.01
Diabetes	37 (41)	16 (38)	299 (27)	.01
Heart failure	22 (24)	0 (0)	35 (3)	<.005
Peripheral vascular disease	20 (22)	1 (2)	58 (5)	<.005
Coronary artery disease	14 (15)	4 (10)	60 (5)	<.005
Chronic respiratory disease	31 (33)	7 (17)	211 (19)	<.005
Chronic renal disease	33 (35)	7 (17)	157 (14)	<.005
Previous stroke/TIA	12 (13)	0 (0)	35 (3)	<.005
CHA_2_DS_2_-VASc score				
0	3 (3)	7 (17)	304 (28)	<.005
1	6 (6)	9 (21)	354 (32)	
>1	85 (91)	26 (62)	447 (40)	
Premorbid state				<.005
Independent	44 (48)	39 (93)	936 (85)	
POC	38 (41)	1 (2)	88 (8)	
Residential home	0 (0)	0 (0)	7 (1)	
Nursing home	12 (13)	2 (5)	67 (6)	
Unknown	0 (0)	0 (0)	7 (1)	
Anticoagulation status preadmission				<.005
None	11 (13)	32 (76)	897 (81)	
Antiplatelets	15 (16)	9 (21)	136 (12)	
Prophylactic LMWH	1 (1)	1 (2)	8 (1)	
Warfarin/DOAC/treatment dose LMWH	67 (72)	0 (0)	54 (5)	
Unknown	0 (0)	0 (0)	10 (1)	

Values are range or n (%).

Values are *P* values represent those calculated by Kruskal-Wallis test.

CHA_2_DS_2_-VASc = congestive heart failure, hypertension, age ≥75 years, diabetes mellitus, prior stroke or transient ischemic attack or thromboembolism, vascular disease, age 65–74 years, sex category; DOAC = direct oral anticoagulant; LMWH = low-molecular-weight heparin; POC = package of care; TIA = transient ischemic attack.

### COVID-19 and new-onset AF

AF was the most common arrhythmia in patients with COVID-19 (Table 2). During their hospital stay, 42 (3.3%) patients were diagnosed with new-onset AF, of whom 30 (71%) required intensive care admission.

**Table 2:** Subclassification of arrhythmia complications in patients admitted with COVID-19 infection

Arrhythmia subtype	No. of patients (%)
Atrial fibrillation	42 (3.4)
Bradyarrhythmia	14 (1.1)
Supraventricular tachycardia	8 (0.6)
Ventricular tachycardia	6 (0.5)
Atrial flutter	3 (0.2)

**Figure 2:**
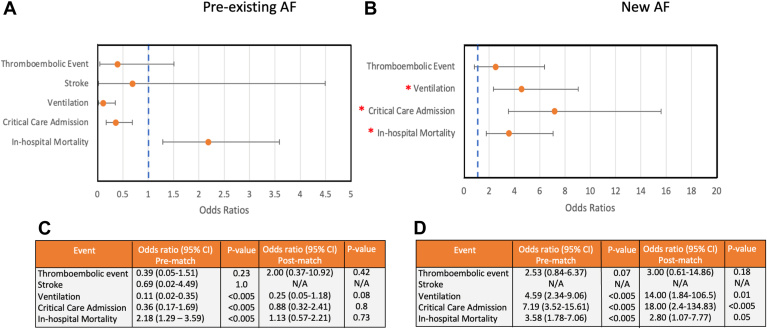
Forest plots and tabulation representing prematch and postmatch odds ratios for thromboembolic event, ischemic stroke, ventilation, critical care admission, and in-hospital mortality in patients with pre-existing atrial fibrillation (AF) (A, C) and new AF (B, D) compared with patients with no AF. CI = confidence interval; N/A = not applicable.

New-onset AF was associated with an increased risk of mechanical ventilation (odds ratio [OR] 4.59, 95% confidence interval [CI] 2.34-9.06, *P <* .005) and intensive care admission (OR 7.19, 95% CI 3.52-15.61, *P <* .005) prior to propensity sore matching. Statistical significance remained after propensity score matching (mechanical ventilation: OR 14.00, 95% CI 1.84-106.5, *P =* .01; intensive care admission: OR 18, 95% CI 2.40-134.83, *P <* .005). In-hospital mortality was more likely in patients with new AF (OR 3.58, 95% CI 1.78-7.06, *P <* .005) compared with patients with no known AF and remained elevated after adjustment for age, sex, race and preadmission CHA_2_DS_2_-VASc score (OR 2.80, 95% CI 1.01-7.77, *P =* .048) (Figure 2).

New-onset AF was associated with older age (*P <* .005), higher CHA_2_DS_2_-VASc score (*P <* .005), elevated white cell count (*P =* .046), elevated neutrophil count (*P =* .010), elevated C-reactive protein (*P <* .005), elevated ferritin (*P =* .020), lower albumin (*P <* .005), and lower eGFR (*P =* .013) at the time of hospital admission. No association was found with sex (*P =* .683) or race (*P =* .080).

**Figure 3:**
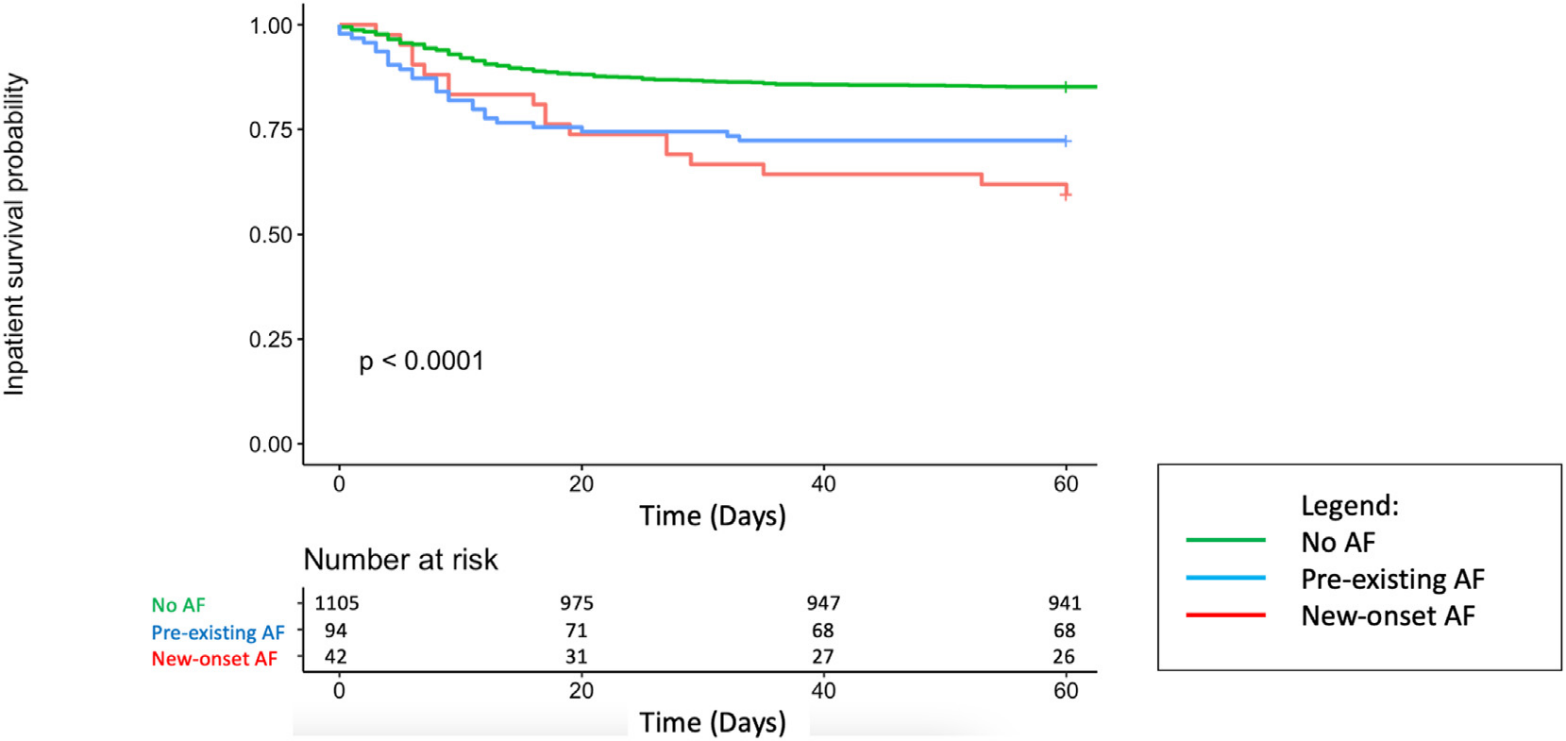
Kaplan-Meier Curve for all-cause mortality in patients with new-onset atrial fibrillation (AF), pre-existing AF, and no AF demonstrating reduced inpatient survival in the new-onset AF group compared with the no-AF group.

There was a statistically significant difference in survival distributions between patients stratified by AF classification (Figure 3). Inpatient survival probability was highest in the no-AF group and lowest in the new-onset AF group. Following pairwise comparison, statistical significance was found between pre-existing AF and no-AF groups (*P <* .005) and new AF and no-AF groups (*P <* .005). There was no statistical significance between survival distributions in patients with pre-existing AF and new AF (*P =* .723).

## COVID-19 and pre-existing atrial fibrillation

**Figure 4:**
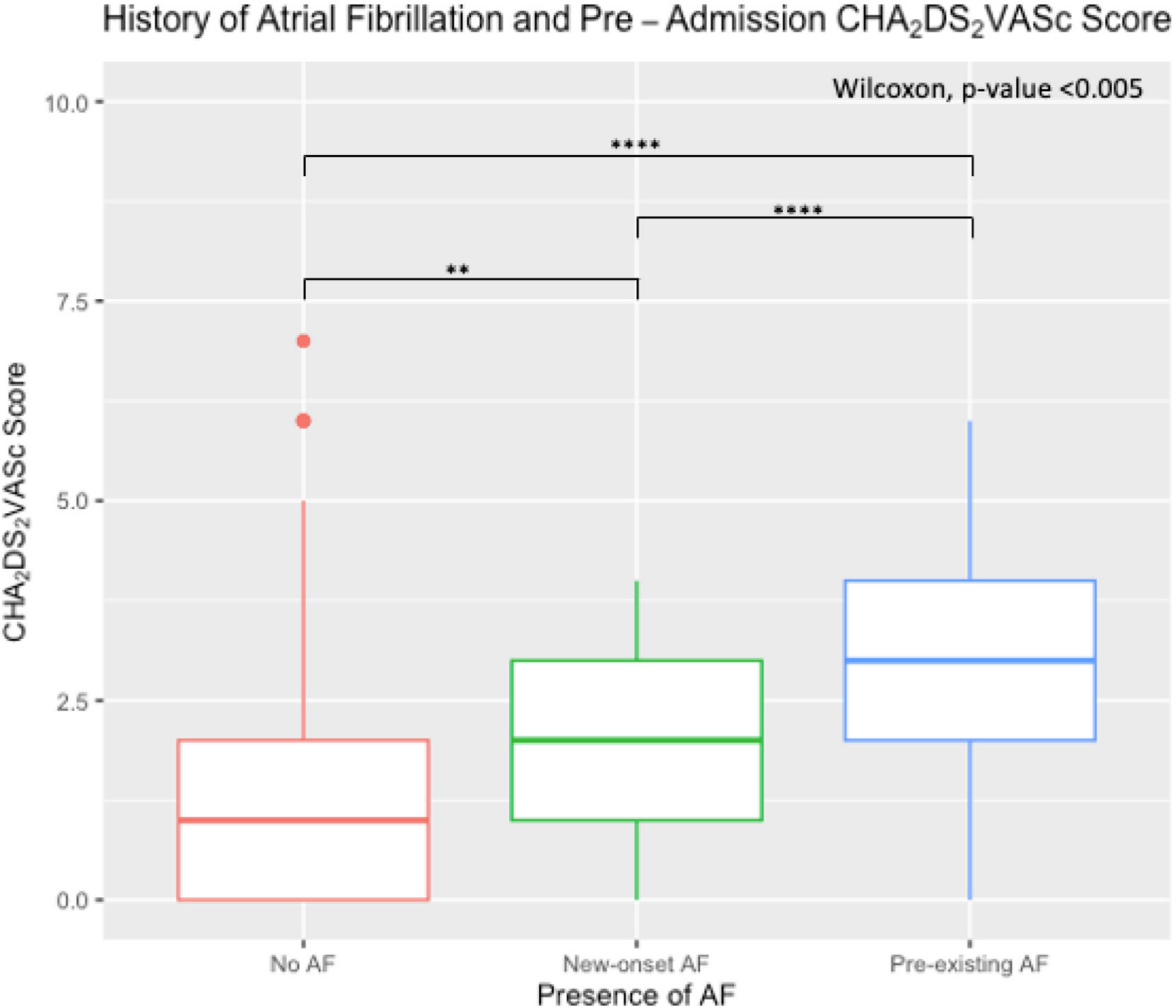
History of atrial fibrillation (AF)and preadmission CHA_2_DS_2_-VASc (congestive heart failure, hypertension, age ≥75 years, diabetes mellitus, prior stroke or transient ischemic attack or thromboembolism, vascular disease, age 65–74 years, sex category) score. Increased preadmission CHA_2_DS_2_-VAScscore between no AF and new-onset AF, new-onset AF and pre-existing AF, and no AF and pre-existing AF (*P* < .05).

A total of 94 (7.6%) patients hospitalized with COVID-19 had pre-existing AF. The median CHA_2_DS_2_-VASc score in patients with pre-existing AF was 3 (IQR 2-4) compared with 1 (IQR 0-2) in the new-onset AF and no-AF groups (*P <* .05) (Figure 4). Pre-existing AF was also associated with additional comorbidities including hypertension, diabetes and coronary artery disease (*P* = .01) (Table 1). Of the patients with pre-existing AF, 71% were receiving anticoagulation therapy prior to hospital admission.

In univariate analysis including all patients from the original dataset, the odds of in-hospital mortality were twice as likely in patients with pre-existing AF compared with patients with no AF (OR 2.18, 95% CI 1.29–3.59, *P =* .002). After propensity score matching, there was no statistically significant difference in the primary outcome between patients with pre-existing AF and patients with no AF (OR 1.13, 95% CI 0.57–2.21, *P =* .732) (Figure 2).

Pre-existing AF was associated with a reduced risk of mechanical ventilation (OR 0.11, 95% CI 0.02–0.35, *P <* .005) and intensive care admission (OR 0.36, 95% CI 0.17–1.69, *P <* .005) prior to propensity sore matching. However, there was no statistical significance after propensity score matching (mechanical ventilation: OR 0.25, 95% CI 0.05–1.18, *P =* .08; intensive care admission: OR 0.88, 95% CI 0.32–2.41, *P =* .80).

## Discussion

This is the largest study to date using manual chart review to study the relationship between sinus rhythm, new-onset AF, pre-existing AF and clinical outcomes in patients with COVID-19. In contrast to previous studies, the size of this study allowed a matched analysis to be performed between cohorts to reduce the effect of confounding variables. We demonstrate that, in patients hospitalized with COVID-19, new-onset AF is the most common cardiac arrhythmia complication and is associated with an increased risk of all-cause in-hospital mortality, need for mechanical ventilation, and critical care admission. In contrast, while pre-existing AF is associated with greater prevalence of comorbidities in hospitalized COVID-19 patients, it is not independently associated with all-cause in-hospital mortality after adjusting for age, sex, race, and preadmission CHA_2_DS_2_-VASc score.

## Data curation

Manual curation of data allows for verification of data, improved data accuracy, and a reduction in missing data, challenges that are common when using large registry datasets and automatic methods of data collection.^17^ Difficulties in manual curation of large datasets mainly exist due to labor time in the context of limited resources. Larger studies assessing clinical outcomes of patients with COVID-19 and AF have therefore often been performed using automatic extraction from electronic healthcare records or registry datasets.^5^^,^^10^^,^^13^^,^^14^ These techniques have been shown to miss important clinical features. Indeed, in an automated study of 3970 patients with COVID-19, manual review of clinical records in a subset of 1110 patients was found to capture a higher incidence of AF/atrial flutter and prevalence of comorbidities compared with automatic extraction from electronic healthcare records. This highlights significant ongoing limitations of automatic data collection.^10^ In the largest study to date of employing automatic data curation methods, AF was diagnosed in 1687 of 9564 COVID-19 patients using natural language processing techniques and found to be an independent predictor of in-hospital mortality.^14^ However, no manual validation was performed in this study. While allowing rapid assimilation of large quantities of data, machine learning techniques such as natural language processing are reliant on correct inference of electronic health record notes, which remains a key challenge in such processing techniques.^18^ In contrast to automatic methods of data collection, data in this study were manually curated for improved accuracy and data verification, ensuring great confidence in the data obtained.^17^

### COVID and pre-existing atrial fibrillation

In line with large-scale population-based studies, patients with COVID-19 and pre-existing AF were more likely to be older, have increased frailty, and have pre-existing respiratory or renal disease compared with patients with new-onset AF and no AF.^19^ They were significantly more likely to have additional vascular risk factors including hypertension, diabetes, heart failure, peripheral vascular disease, and coronary artery disease. These data therefore suggest that pre-existing AF is a surrogate marker for morbidity status, rather than an independent marker of mortality in COVID-19. This observation accounts for the present study findings of a statistically significant increase in the risk of in-hospital mortality of patients with pre-existing AF in unadjusted analysis, yet a nonsignificant finding after propensity score matching.

Furthermore, lower baseline functional state and increased morbidity in this cohort may have resulted in reduced admissions to intensive care and invasive ventilation compared with patients with new-onset AF due to clinical recommendations and preagreed restrictions on appropriate ceiling of care and resuscitation status. This may provide an explanation for pre-existing AF being protective against intubation and ventilation in COVID-19 infection in unadjusted analysis.

### COVID and new-onset AF

In contrast to patients with pre-existing AF, patients with new-onset AF were younger, with fewer comorbidities and a lower CHA_2_DS_2_-VASc score. Furthermore, hypertension and diabetes in particular were more common in the new-onset AF group compared with the sinus rhythm group. It is well established that hypertension and diabetes have specific effects on atrial structure and electrophysiological function, and these effects are frequently documented in experimental models in the absence of sustained AF. As such, it is feasible that the new-onset AF group may highlight a group of patients that are susceptible to AF, which becomes clinically apparent during severe COVID-19 infection.

In keeping with the findings of this study, previous studies have noted an increase in markers of disease severity and need for intensive care admission in new-onset AF patients compared with pre-existing AF and sinus rhythm patients.^13^^,^^14^ Smaller studies have provided conflicting data on whether new-onset AF is an independent markers of disease severity and all-cause mortality in patients with COVID-19. Both Russo and colleagues ^7^ and Sanz and colleagues^12^ demonstrated no difference in acute respiratory syndrome or all-cause mortality in patients with new-onset AF compared with those with no AF.Click or tap here to enter text. However, Sano and colleagues^13^ demonstrated significantly worse outcomes in patients with new-onset AF compared with patients in sinus rhythm or those with pre-existing AF. In this study, which is currently the largest manually curated dataset, new-onset AF was found to be independently associated with increased need for mechanical ventilation, critical care admission, and inpatient mortality. The time of AF onset is unknown, as these data were not collected during this study, and therefore it is unclear whether the development of new-onset AF is an early or late marker of severe COVID-19. Further research is needed to investigate whether new-onset AF predicts future clinical deterioration.

In the present study, AF was the most common cardiac arrhythmia present. This finding has also been observed in several other studies in which the prevalence of new-onset AF ranged from 3.5% to 7.5%.^12^^,^^20^, ^21^, ^22^ While previous studies have reported worse outcomes in patients with AF and COVID-19, this study is the first to disentangle the relationship between outcomes in patients with pre-existing AF and new-onset AF with the analysis certainty brought by manual chart review. This study demonstrates that new-onset AF but not pre-existing AF is independently associated with in-hospital mortality. This is of particular importance because only new-onset AF can be a direct consequence of COVID-19 infection. Previous studies have suggested that COVID-19 may have cardiotoxic effects via direct and indirect mechanisms and new-onset AF may therefore be a specific marker of cardiac injury resulting in poorer outcomes.^23^ Recent data indicate that cardiovascular complications of COVID-19 continue to occur following COVID-19 infection.^24^ Although the specific pathophysiology of this remains under investigation and is likely multifaceted, possible mechanisms include the effects of COVID-19 infection on angiotensin-converting enzyme 2–related signaling pathways, cytokine storm, changes in fluid balance, hypokalemia, hypoxemia, and activation of the sympathetic nervous system.^23^^,^^25^^,^^26^ However, several of these mechanisms are not specific to COVID-19 infection but can be attributed to the physiological response to critical illness. It is recognized that non-COVID acute respiratory viral infection requiring critical care admission is associated with an increased incidence of new-onset AF.^26^ The presence of new-onset AF may therefore be a marker of disease severity rather than a specific consequence of COVID-19 infection, although this requires further investigation. Nevertheless, patients diagnosed with new-onset AF in the context of COVID-19 should be monitored closely for acute deterioration and need for advanced care.

### Limitations

Although this was a single-center study, the population served by our institution is diverse as reflected by the demographic and ethnicity variability in the study population. This study included patients hospitalized and therefore excludes asymptomatic patients or those with mild COVID-19 symptoms. Furthermore, patients were not followed up beyond their hospital stay and clinical outcomes therefore represent the acute phase of COVID-19.^27.^ COVID polymerase chain reaction testing was used to determine COVID status, and while there may be false positive or negative results, it remains the gold standard diagnostic investigation for COVID-19 infection. Finally, new-onset AF patients were defined as such if there was no known history of AF within the community. Without continuous heart rhythm within the community, it is feasible that some of these patients may have had asymptomatic pre-existing AF. Of the 45 patients categorized in the new-onset AF group, 10 patients had historical electrocardiograms that confirmed sinus rhythm prior to admission. While we acknowledge this does not exclude a history of paroxysmal AF, it is noted that this is a frequent limitation present in all large population-based AF studies including the Framingham Heart Study and more recently the Nationwide Finnish anticoagulation in atrial fibrillation study.^27^^,^^28^ The results of this study are therefore interpretable through the same lens as this large body of prior literature.

## Conclusion

In patients hospitalized with COVID-19, new-onset atrial fibrillation is independently associated with elevated risk of need for mechanical ventilation, critical care admission, and in-hospital mortality. In contrast, while pre-existing AF is associated with greater prevalence of comorbidities in hospitalized COVID-19 patients, it is not independently associated with all-cause in-hospital mortality after adjusting for age, sex, race, and preadmission CHA_2_DS_2_-VASc score. Patients with new-onset AF in the context of COVID-19 should be closely monitored for acute deterioration and need for escalation of care. This study highlights the need for targeted research to explain the mechanistic relationship between new-onset atrial fibrillation and COVID-19.
